# The first complete chloroplast genome in *Engelhardia sensu stricto*, *Engelhardia hainanensis* Chen: genome characterization and its phylogenetic relationships within the family Juglandaceae

**DOI:** 10.1080/23802359.2023.2196359

**Published:** 2023-04-10

**Authors:** Yuan-Mi Wu, Xian-Yun Mu, Yong-Hua Qin

**Affiliations:** a Laboratory of Systematic Evolution and Biogeography of Woody Plants, College of Ecology and Nature Conservation, Beijing Forestry University, Beijing, P. R. China; b Guangxi Forestry Inventory and Planning Institute, Nanning, P. R. China

**Keywords:** *Alfaropsis*, complete chloroplast genome, *Engelhardia hainanensis*, Juglandaceae

## Abstract

Trees of *Engelhardia* are important components of subtropical and tropical forests in South-eastern Asia with great ecological and economic values. However, phylogenetic relationships within Engelhardioideae (Juglandaceae) remains obscure. In this study, we report the first complete chloroplast genome sequences of *Engelhardia sensu stricto*, *Engelhardia hainanensis* Chen, a rare species endemic in southern China. Its complete chloroplast genome is 161,574 bp in length, with a typical quadripartite structure that includes a large single-copy region of 91,158 bp, a small single-copy region of 18,790 bp, and its GC content is 35.8%. A total of 128 genes were identified, including 83 protein-coding genes, 37 tRNA genes, and 8 rRNA genes. Furthermore, a phylogenetic tree of Juglandaceae was constructed based the complete chloroplast genome sequence, which strongly support the three-subfamily classification system in Juglandaceae, and *E. hainanensis* was resolved sister to two *Alfaropsis* species. This study provides valuable genomic information for the species identification and phylogenetic study of Juglandaceae.

## Introduction

**Figure 1.**
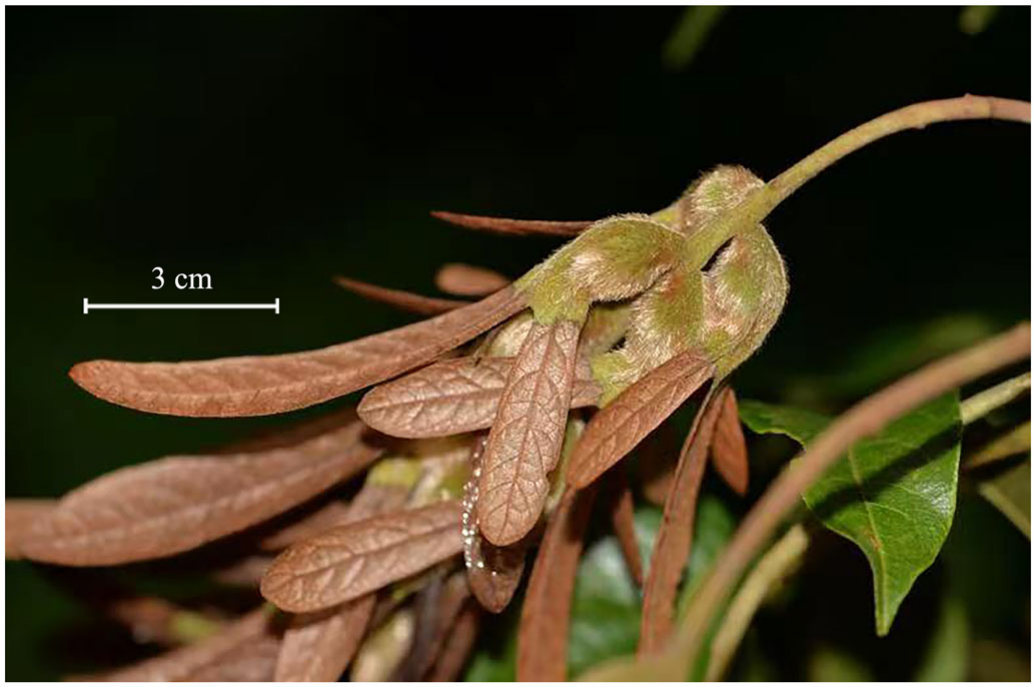
Fruits with winged bracts of *Engelhardia hainanensis*. This photo was photographed by XYM at Lingyun County in Guangxi.

*Engelhardia* Leschenault ex Blume is a deciduous, semievergreen or evergreen tree group of Juglandaceae which distributes in South-Eastern Asia from eastern Pakistan to New Guinea (Lu et al. 1999). Members of this group are important component in the subtropical and tropical forests, whose leaves are used for drinking as tea while its bark are used to poison fish in local community. Contrary to great ecological and economic value, the subgeneric classification and species number of *Engelhardia* is open to question (Meng et al. 2022). *Engelhardia* is suggested divided into genus *Engelhardia sensu stricto* (*s.s.*) which contains ca. five species, and a monotypic genus *Alfaropsis* Iljinsk. which contains only *Alfaropsis roxburghiana* (Wall.) Iljinsk. (Iljinskaya 1993). A distinct morphological difference between *Engelhardia s.s.* and *Alfaropsis* lies in that prophyllum is obvious and envelops fruit in the former while it is absent in the latter. *Engelhardia hainanensis* Chen (Chen 1981) is a rare species which has the longest winged bracts out of fruit (ca. 8cm, Figure 1) in the genus, and it is thought endemic in Hainan Island, China. However, we discover it in Guangxi Zhuang Autonomous Region during our recent field investigation. In order to better understand the phylogenetic relationships within *Engelhardia* and Juglandaceae, and provide more genomic data for accurate identification of the toxic *Engelhardia* species, we sequenced and analyzed the first complete chloroplast genome of *Engelhardia s.s.*, *E. hainanensis*.

## Materials and methods

### Sample collection and preservation

The fresh leaves of *E. hainanensis* were collected at Lingyun County in Guangxi, China (N24°31′52.86″, E106°27′43.67″). Leaves were dried with silica gel in the field. Once completely dried, the leaf tissues were stored at −20 °C freezer until further use. Voucher specimen (collector and collection number: Xian-Yun Mu (xymu85@bjfu.edu.cn) and MU4973) is deposited at the herbarium of Beijing Forestry University (www.bjfu.edu.cn).

### DNA extraction and sequencing, and cleaning raw reads

Genomic DNA were extracted from the above leaf tissues using the DNAsecure Plant Kit (Tiangen Biotech Co. Ltd., Beijing, China), and sequenced by next-generation sequencing method on Illumina Hiseq X Ten platform. In total of 4.1 Gb of 150-bp clean reads were generated for chloroplast genome assembly.

### Assembly, annotation, and visualization

Complete chloroplast genome assembly was performed on GetOrganelle 1.7.5 (Jin et al. 2020), the annotation was performed on CPGAVAS2 (Shi et al. 2019), and further verification by Geneious Prime 2022 (Kearse et al. 2012) with *A. roxburghiana* (Ling and Zhang 2020) as a reference. The complete plastome of *E. hainanensis* has been deposited in GenBank under accession number OM302449. A circular map of its plastome was visualized using the CPGView online web (http://www.1kmpg.cn/cpgview, Liu et al. 2023)

### Phylogenetic reconstruction

To determine the phylogenetic relationships of *E. hainanensis* in Juglandaceae, 40 species in Juglandaceae and three species in Betulaceae, Fagaceae, and Myricaceae family were selected based on previous researches (e.g., Zhang et al. 2019; Mu et al. 2020; Song et al. 2020). These related plastome sequences were downloaded from NCBI (https://www.ncbi.nlm.nih.gov/). All sequences were aligned using MAFFT (Katoh et al. 2019). A maximum likelihood tree with bootstrap value (1000 replicates) was constructed based on GTR + G model in RAxML software (Kozlov et al. 2019). The final tree was edited using the iTOL version 5.0 online web (https://itol.embl.de/) (Letunic and Bork 2016).

## Results and discussion

### Characterization of the chloroplast genome

**Figure 2.**
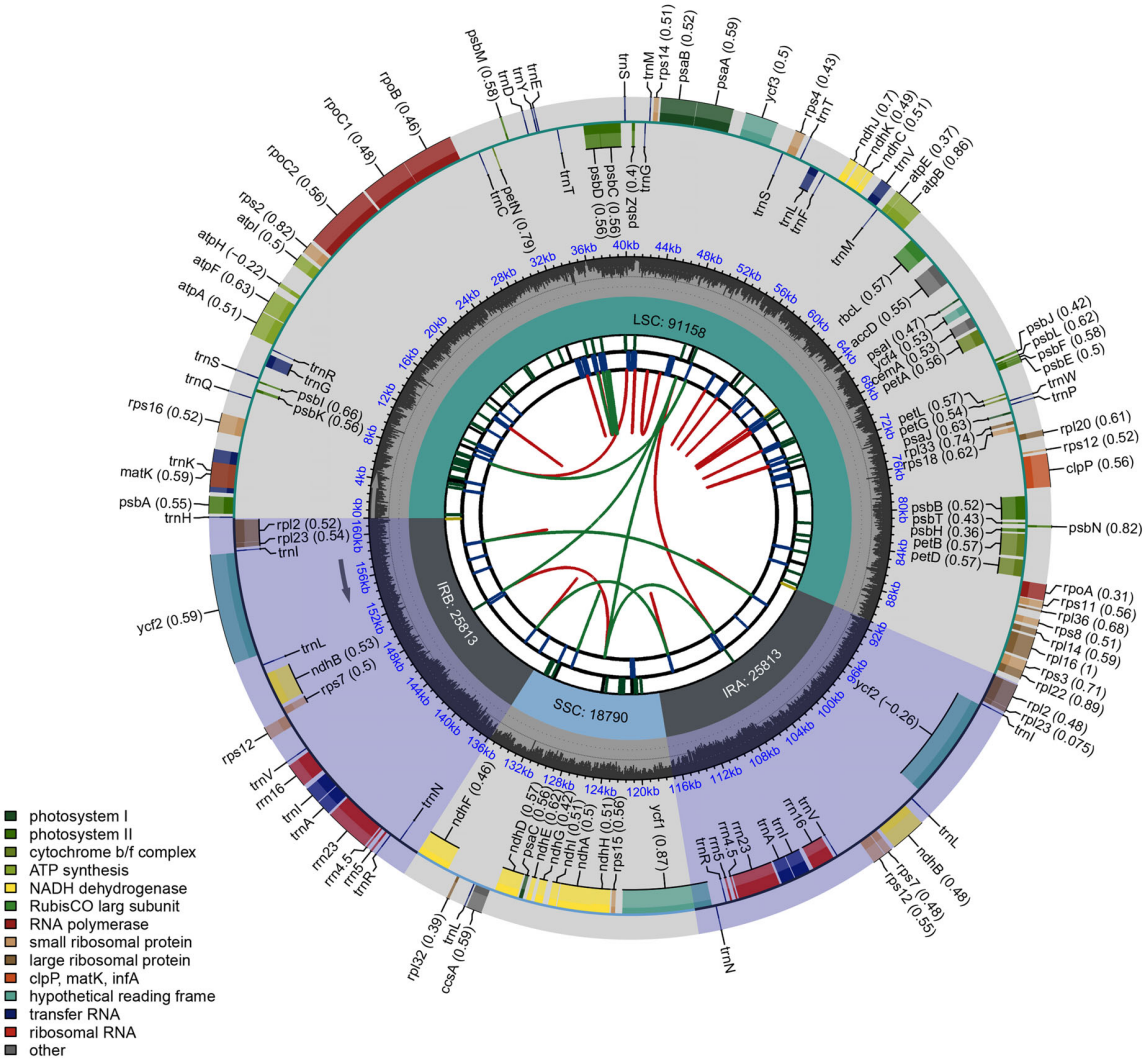
Schematic circular map of overall features of *Engelhardia hainanensis* chloroplast genome. Graphic showing features of its plastome was generated using CPGview. The map contains six tracks. From the inner circle, the first track depicts the dispersed repeats connected by red (forward direction) and green (reverse direction) arcs, respectively. The second track shows the long tandem repeats as short blue bars. The third track displays the short tandem repeats or microsatellite sequences as short bars with different colors. The fourth track depicts the sizes of the inverted repeats (IRa and IRb), small single-copy (SSC), and large single-copy (LSC). The fifth track plots the distribution of GC contents along the plastome. The sixth track displays the genes belonging to different functional groups with different colored boxes. The outer and inner genes are transcribed in the clockwise and counterclockwise directions, respectively.

The complete chloroplast genome of *E. hainanensis* was assembled correctly with 161,574 bp in length (Figure S1), and its schematic circular map was showed in Figure 2. The genome has a typical quadripartite structure, including a large single-copy (LSC) region of 91,158 bp, a small single-copy (SSC) region of 18,790 bp, and two inverted repeat (IRa and IRb) regions of 25,813 bp for each. Overall, the average GC content was 35.8%. A total of 128 genes (110 unique genes) were identified in the chloroplast genome of *E. hainanensis*, including 83 protein-coding genes (77 unique genes), 37 tRNA genes (29 unique genes), and 8 rRNA genes (4 unique genes). Among them, there were 12 protein-coding genes (*rps*16, *atp*F, *rpo*C1, *ycf*3, *clp*P, *pet*B, *pet*D, *rpl*16, *rpl*2, *ndh*B, *ndh*A, *rps*12, Figure S2) and 6 tRNA genes (*trn*K-UUU, *trn*G-UCC, *trn*L-UAA, *trn*V-UAC, *trn*I-GAU, *trn*A-UGC) with introns. The trans-splicing gene *rps*12 had three unique exons (Figure S3).

### Phylogenetic analysis of E. hainanensis within the family Juglandaceae

**Figure 3.**
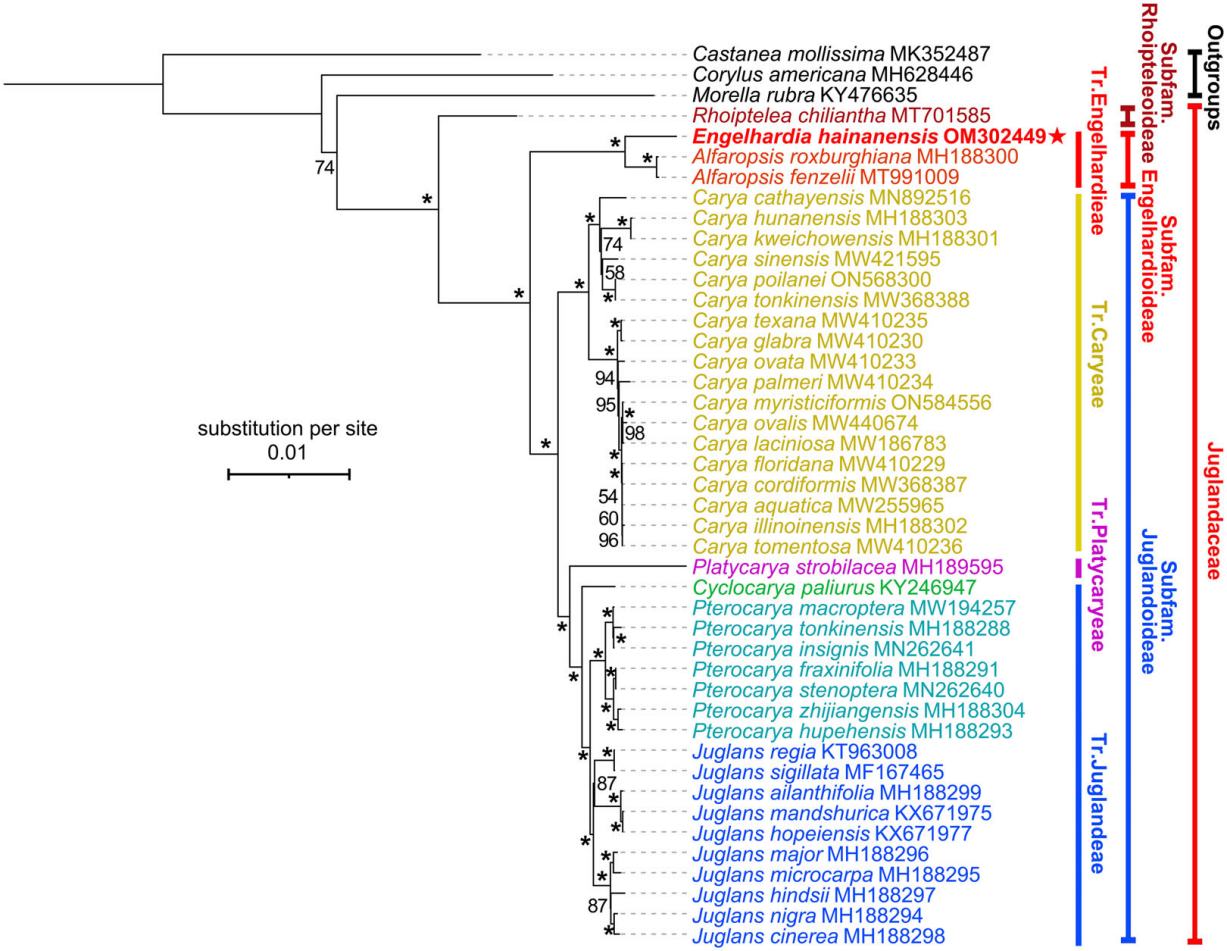
Phylogenetic tree of Juglandaceae inferred from maximum-likelihood (ML) method based on concatenated complete chloroplast genome sequence of 41 species with *Corylus americana*, *Castanea mollissima*, *and Morella rubra* as outgroup. *The clade support value of 100 that was generated from the maximum likelihood method. The red star represents the assembled plastome sequence (i.e., *Engelhardia hainanensis* OM302449) in this study. GenBank accession numbers of the following sequences were used: *Castanea mollissima* MK352487 (Zhu et al. 2019), *Corylus americana* MH628446 (Zhao et al. 2020), *Morella rubra* KY476635 (Liu et al. 2017), *Rhoiptelea chiliantha* MT701585 (Jin et al. 2020), *Alfaropsis roxburghiana* MH188300 (Zhou et al. 2021), *Alfaropsis fenzelii* MT991009 (Liu et al. 2021), *Carya cathayensis* MN892516 (Shen et al. 2022), *Carya hunanensis* MH188303 (Zhou et al. 2021), *Carya kweichowensis* MH188301 (Zhou et al. 2021), *Carya sinensis* MW421595 (Xi et al. 2022), *Carya poilanei* ON568300 (Xi et al. 2022), *Carya tonkinensis* MW368388 (Xi et al. 2022), *Carya texana* MW410235 (Xi et al. 2022), *Carya glabra* MW410230 (Xi et al. 2022), *Carya ovata* MW410233 (Xi et al. 2022), *Carya palmeri* MW410234 (Xi et al. 2022), *Carya myristiciformis* ON584556 (unpublished), *Carya ovalis* MW410234 (Xi et al. 2022), *Carya laciniosa* MW186783 (Zhai et al. 2021), *Carya floridana* MW410229 (Xi et al. 2022), *Carya cordiformis* MW368387 (Xi et al. 2022), *Carya aquatica* MW255965 (Xi et al. 2022), *Carya illinoinensis* MH188302 (Zhou et al. 2021), *Carya tomentosa* MW410236 (Xi et al. 2022), *Platycarya strobilacea* MH189595 (Zhou et al. 2021), *Cyclocarya paliurus* KY246947 (Hu et al. 2017), *Pterocarya macroptera* MW194257 (Yan et al. 2021), *Pterocarya tonkinensis* MH188288 (Zhou et al. 2021), *Pterocarya insignis* MN262641 (Mu et al. 2020), *Pterocarya fraxinifolia* MH188291 (Zhou et al. 2021), *Pterocarya stenoptera* MN262640 (Mu et al. 2020), *Pterocarya zhijiangensis* MH188304 (Zhou et al. 2021), *Pterocarya hupehensis* MH188293 (Zhou et al. 2021), *Juglans regia* KT963008 (Hu et al. 2017), *Juglans sigillata* MF167465 (Dong et al. 2017), *Juglans ailanthifolia* MH188299 (Zhou et al. 2021), *Juglans mandshurica* KX671975 (Hu et al. 2017), *Juglans hopeiensis* KX671977 (Hu et al. 2017), *Juglans major* MH188296 (Zhou et al. 2021), *Juglans microcarpa* MH188295 (Zhou et al. 2021), *Juglans hindsii* MH188297 (Zhou et al. 2021), *Juglans nigra* MH188294 (Zhou et al. 2021), *Juglans cinerea* MH188298 (Zhou et al. 2021).

Phylogenetic relationships intra Juglandoideae are frequently investigated (Zhang et al. 2019; Mu et al. 2020; Song et al. 2020), while those within Engelhardioideae are obscure (Zhang et al. 2020). We reconstructed the phylogeny of Juglandaceae based on whole chloroplast genome sequences of 40 reported species and the new data in this study, and *Castanea mollissima* Blume (Blume 1851) (Fagaceae), *Corylus americana* Walter (Walter 1788) (Betulaceae), and *Morella rubra* Lour. (Loureiro 1790) (Myricaceae) were selected as outgroups. Three main clades are recovered with full support values, which corresponding to the three subfamilies of Juglandaceae, i.e., subfamily Rhoipteleoideae, subfamily Engelhardioideae, and subfamily Juglandoideae (Figure 3). This is consistent with previous phylogenetic analyses (e.g., Zhang et al. 2019; Mu et al. 2020; Song et al. 2020). Further, *E. hainanensis* was resolved a sister clade to the two *Alfaropsis* species (i.e., *A. roxburghiana* and *A. fenzelii*). A large sampling scheme covering more species is needed for further clarification of phylogenetic relationships intra subfamily Engelhardioideae.

## Conclusions

We reported the first complete chloroplast genome sequences of *Engelhardia s.s.*, *E. hainanensis*. The assembly circular plastome was 16,534 bp in length. The phylogenetic analysis results strongly supported the three-subfamily classification system in Juglandaceae, and *E. hainanensis* was a resolved sister to two *Alfaropsis* species. The plastome sequence of *E. hainanensis* presented here provides valuable genomic information for further species identification and phylogenetic study of Juglandaceae.

## Supplementary Material

Supplemental Material

Supplemental Material

Supplemental Material

## Data Availability

The genome sequence data that support the findings of this study are openly available in GenBank of NCBI at (https://www.ncbi.nlm.nih.gov/) under the accession no. OM302449. The associated BioProject, SRA, and BioSample numbers are PRJNA797548, SRP355546 and SAMN25010399, respectively.
