# Supplementary figures and images for: The first complete chloroplast genome in Engelhardia sensu stricto, Engelhardia hainanensis Chen: genome characterization and its phylogenetic relationships within the family Juglandaceae
Source: Mitochondrial DNA B Resour. 2023 Apr 10;8(4):479–83. doi: 10.1080/23802359.2023.2196359 (PMC10101671; doi:10.1080/23802359.2023.2196359)

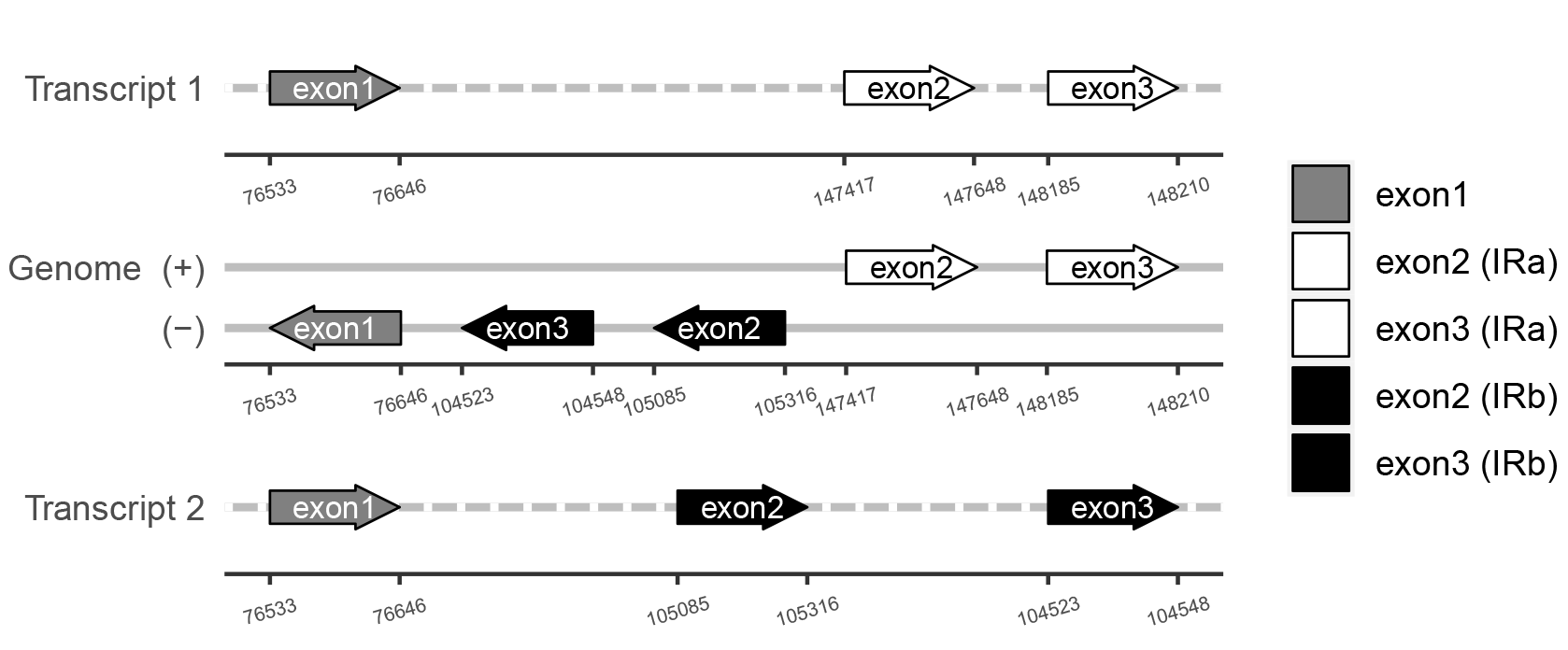

Supplement: Supplemental Material [file TMDN_A_2196359_SM7397.tif]

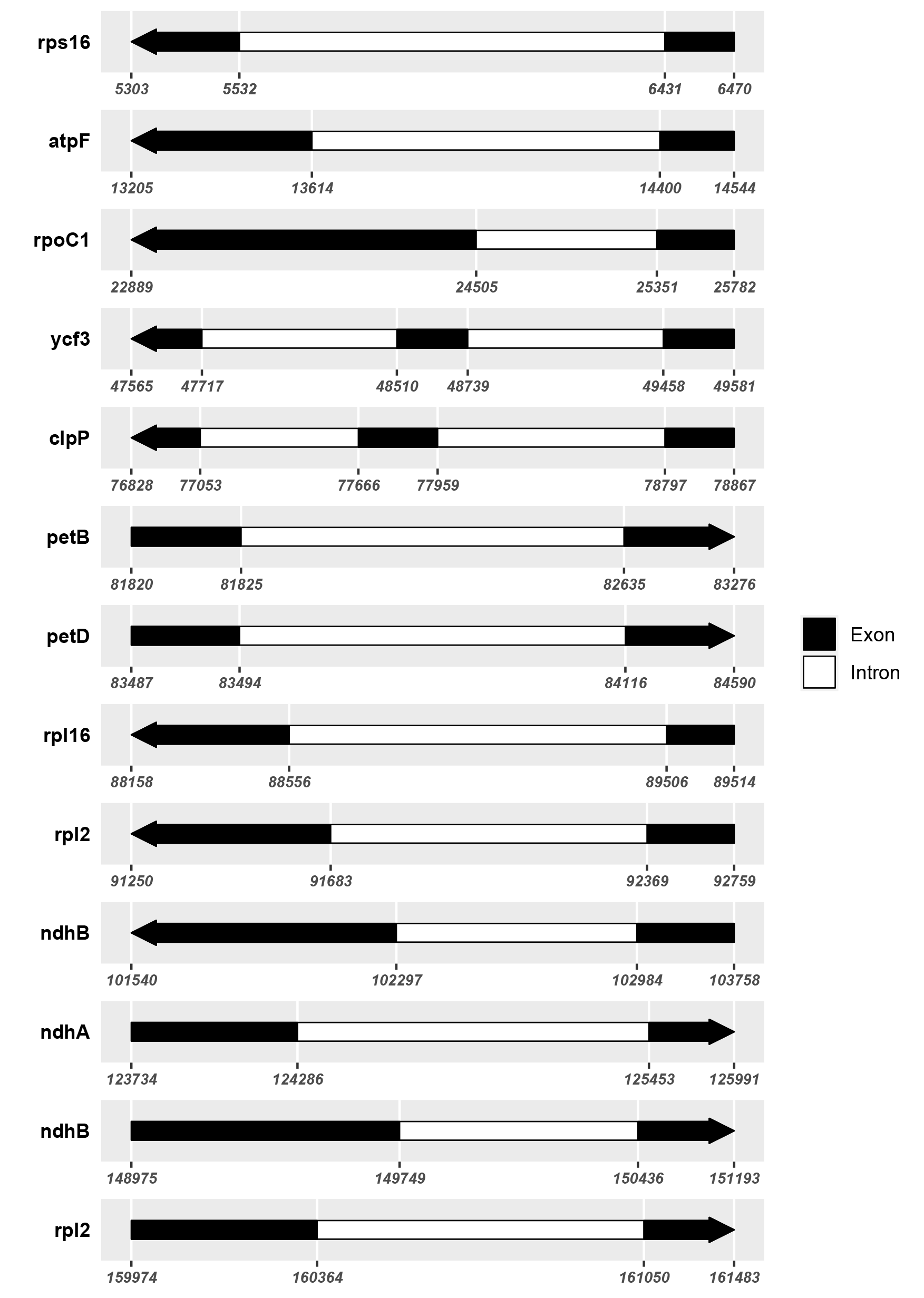

Supplement: Supplemental Material [file TMDN_A_2196359_SM7354.tif]

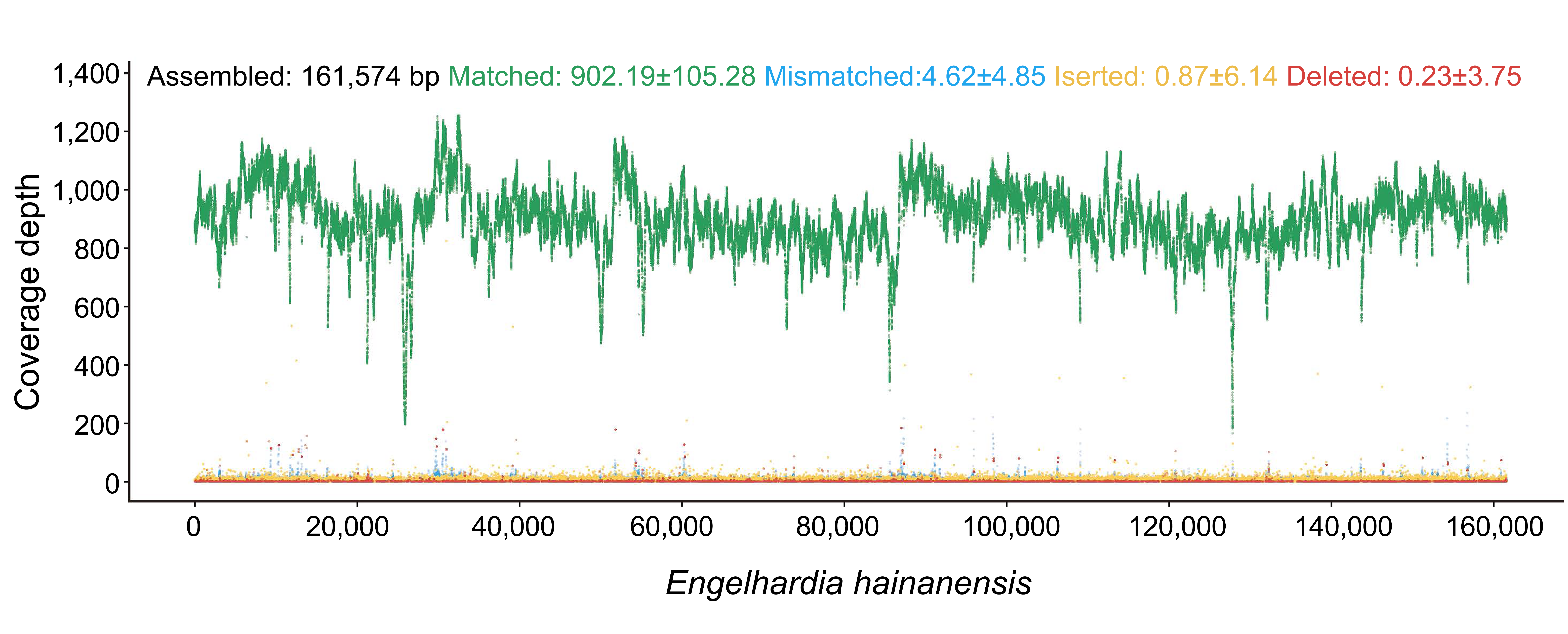

Supplement: Supplemental Material [file TMDN_A_2196359_SM7109.tif]
